# Estimating myocardial fibrosis in aortic stenosis using the serum collagen type I C‐terminal telopeptide to matrix metalloproteinase‐1 ratio

**DOI:** 10.1002/mco2.70069

**Published:** 2025-01-16

**Authors:** Svante Gersch, Philipp Bengel, Niels B. Paul, Susana Ravassa, Stephan von Haehling, Andreas Fischer, Elisabeth M. Zeisberg, Miriam Puls, Gerd Hasenfuß, Moritz Schnelle

**Affiliations:** ^1^ Department of Clinical Chemistry Department of Cardiology and Pneumology University Medical Center Göttingen Göttingen Germany; ^2^ Program of Cardiovascular Disease Centro de Investigacion Medica Aplicada Universidad de Navarra (CIMA) Pamplona Spain

1

Dear Editor,

Aortic stenosis (AS) is the most prevalent valvular disorder in the western world imposing significant morbidity and mortality. Progressive AS primarily affects the left ventricle, leading to adaptations of the myocardium, resulting in cardiac hypertrophy and myocardial fibrosis (MF). We and others recently demonstrated a link between the amount of MF and impaired outcomes following aortic valve replacement in patients with severe AS,^1^ highlighting its clinical relevance. Although cardiac biopsy remains the gold standard for MF quantification, this method is complex, invasive, and may be associated with severe complications. Thus, easier ways of determining MF levels are direly needed.

Different peptides of collagen metabolism have recently been suggested as serum biomarkers for MF and/or associated risk prediction in patients with heart failure (HF).^2^ For instance, low serum levels of procollagen type I C‐terminal propeptide (PICP), formed during the extracellular conversion of procollagen type I into mature fibril‐forming collagen type I, were shown to be linked to reduced risk of cardiovascular death or HF‐associated hospitalization in HF patients with reduced ejection fraction (EF). Levels of the amino‐terminal propeptide of procollagen type III (PIIINP), formed during the conversion of procollagen type III into mature collagen type III, correlated with the collagen type III volume fraction in HF patients with ischemic heart disease or idiopathic dilated cardiomyopathy.

The level of MF is not only a matter of quantity, that is, collagen deposition, but also quality as reflected by the degree of myocardial collagen cross‐linking. This can be indirectly assessed by the serum collagen type I C‐terminal telopeptide to matrix metalloproteinase‐1 ratio (CITP:MMP1), a negative index of myocardial collagen cross‐linking, and can be used for HF‐associated hospitalization risk prediction in hypertensive HF.^3^

Investigations on serum biomarker identification for MF assessment in AS patients with different hemodynamic subtypes have not yet been performed. Thus, our study was designed to evaluate the association of PICP, PIIINP, and CITP:MMP1 with histological MF (measured as collagen volume fraction) in cardiac biopsies, obtained during transcatheter aortic valve replacement (TAVR), in our well‐defined cohort of patients with severe AS.^1^ Biomarkers were measured in serum samples from 95 AS patients prior to TAVR. This study focused on the four different hemodynamic AS subtypes: (I) normal EF and high transvalvular pressure gradient (NEF‐HG AS), (II) low/reduced EF and high gradient (LEF‐HG AS), (III) low/reduced EF and low gradient referred to as classical low‐flow low‐gradient AS (classical LF‐LG AS), and (IV) paradoxical low‐flow low‐gradient despite normal EF (PLF‐LG AS). Details regarding hemodynamics (Table S1) and a detailed description of material and methods can be found in the Supporting Information.

Of the 95 study participants, 39 belonged to the NEF‐HG AS group, 14 to LEF‐HG AS, 26 to classical LF‐LG AS, and 16 to PLF‐LG‐AS. The mean age of the study cohort was 78.4 years (±6.8), with males constituting 67% of the participants. Cardiovascular risk factors and comorbidities were comparably distributed across the four groups (Table S1). Regarding the analyzed serum biomarker levels, no statistically significant differences across the four groups could be observed (Figure 1A). Upon histological examination of fibrosis and as previously reported,^1^ LEF‐HG AS and classical LF‐LG AS patients displayed a markedly higher MF burden compared to NEF‐HG AS patients (*p* < 0.05) (Figure 1B). Importantly, we discovered a trend for a negative association between the level of histological MF and serum CITP:MMP1 ratios in the cohort as a whole (*r* = −0.18; *p* = 0.08), which could predominantly be attributed to the highly significant association in the classical LF‐LG AS group (*r* = −0.62; *p* = 0.002) (Figure 1C,D). None of the associations with the collagen deposition markers PICP and PIIINP in any of the groups were significant (Figure 1C). Of note, all analyses including serum biomarkers were adjusted for renal function (i.e., estimated glomerular filtration rate), age, and sex.

**FIGURE 1:**
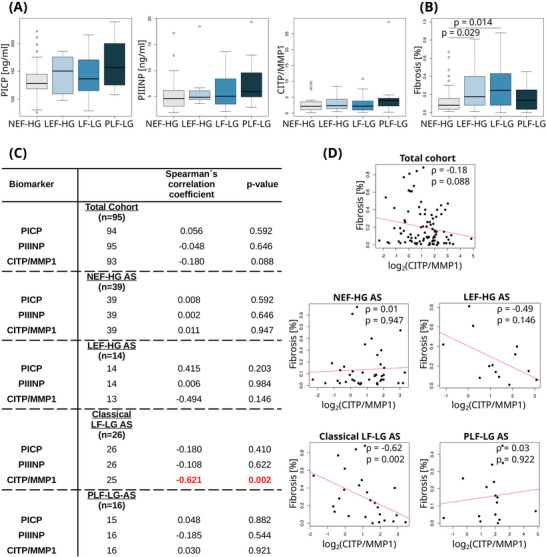
Serum biomarkers of collagen metabolism in different hemodynamic subtypes of severe aortic stenosis. (A) Serum biomarker levels across the four hemodynamic subtypes of aortic stenosis. Box plots show the 25th and 75th (boxes), and 50th (horizontal line) percentile values. (B) Levels of myocardial fibrosis across the four hemodynamic subtypes of aortic stenosis. Box plots show the 25th and 75th (boxes), and 50th (horizontal line) percentile values, while the small circles represent data points outside of this range. *p*‐values of significant differences between groups are also displayed. (C) Association of histologically assessed myocardial fibrosis with serum peptides of collagen metabolism in different hemodynamic subtypes of aortic stenosis. Partial correlations were controlled for age, sex, and the estimated glomerular filtration rate (eGFR). Spearman's correlation coefficients and respective *p*‐values are shown. Significant findings are highlighted in red. (D) Scatterplots showing the percentage of myocardial fibrosis as a function of the log₂‐transformed CITP:MMP1 biomarker ratio for the total cohort and the four subtypes. The redline depicts the least‐squares linear‐regression for the plotted data with *ρ* indicating the partial correlation coefficient and *p* its associated *p*‐value. AS, aortic stenosis; CITP:MMP1, collagen type I C‐terminal telopeptide to matrix metalloproteinase‐1 ratio; NEF‐HG AS, normal ejection fraction and high transvalvular pressure gradient AS; LEF‐HG AS, low/reduced ejection fraction and high transvalvular pressure gradient AS; LF‐LG AS, classical low‐flow low‐gradient AS; PLF‐LG AS, paradoxical low‐flow low‐gradient despite normal ejection fraction AS; PICP, procollagen type I C‐terminal propeptide; PIIINP, amino‐terminal propeptide of procollagen type III.

To our knowledge, this is the first study assessing the three well‐characterized serum biomarkers of collagen metabolism, that is, PICP, PIIINP, and CITP:MMP1, in AS patients with different hemodynamic subtypes. We uncovered a hitherto unrecognized role for CITP:MMP1 as promising serum biomarker for estimating MF levels specifically in classical LF‐LG AS patients. From a pathophysiological perspective, the results point toward myocardial collagen cross‐linking as relevant mechanism specifically in this hemodynamic entity. Collagen cross‐linking plays a critical role in determining the resistance of collagen fibers to MMP‐mediated degradation; thus, enhanced cross‐linking of collagen type I fibers specifically reduces the cleavage of CITP by MMP1. Consequently, the negative association of the CITP:MMP1 ratio with histological MF in classical LF‐LG AS patients is suggestive of more collagen cross‐linking and resistance to degradation, causing reduced cleavage of CITP by MMP1. This results in increased levels of MF and consequently lower CITP:MMP1 serum ratios. It is likely that this mechanism contributes to the adverse myocardial remodeling and worst prognosis of classical LF‐LG AS among all four AS subtypes even after TAVR.^1^ This association could not be observed in any of the other groups, emphasizing the importance to distinguish between them as pathophysiological mechanisms may be fundamentally different. Moreover, our results on PICP and PIIINP suggest that collagen deposition, as compared to cross‐linking, may not be as relevant in our cohort of AS patients, irrespective of the hemodynamic subtype. This could potentially be attributed to the timing of collagen deposition, which may have occurred before the biopsy was taken with no further relevant active collagen production. However—due to the rather small sample size—a type II error, that is, the failure to reject a false null hypothesis, can also not be ruled out at this point.

The relevance to distinguish between hemodynamic AS subgroups is not only important for the identification of novel biomarkers, but also with respect to a more personalized therapeutic strategy. In this regard, assessing CITP:MMP1 serum levels in patients with classical LF‐LG AS may help to identify individuals who particularly benefit from a medication with antifibrotic properties. In line with this consideration, Ravassa et al. already demonstrated the potential of CITP:MMP1 assessment regarding the favorable effects through spironolactone treatment in (i) HF patients with preserved EF (HFpEF) (Aldo‐DHF trial) and (ii) patients at risk of HF (HOMAGE trial).^4^, ^5^ In both studies, patients with higher CITP:MMP1 ratios, that is, a lower degree of collagen cross‐linking, showed various clinical benefits as compared to patients with lower ratios following spironolactone treatment.

Considering the exploratory nature of our study—with its relatively small sample size and primary focus on the correlation between collagen biomarkers and histological fibrosis—larger studies are needed to expand these observations and to evaluate the relationship between CITP:MMP1 and cardiovascular outcome parameters. It will therefore be interesting to assess serum CITP:MMP1 in the ongoing, prospective REDUCE‐MFA trial (NCT05230901). In this cohort, patients with severe AS are treated with low‐dose dihydralazine and spironolactone, aiming to reduce MF. Assessing CITP:MMP1 with subsequent analysis of a potential MF reduction and altered outcome will allow to get a much clearer understanding of how this biomarker could be implemented into clinical practice.

In summary, we identified CITP:MMP1 as a novel serum biomarker for estimating MF in classical low‐flow low‐gradient AS. This is the first study to demonstrate a significant correlation between the serum CITP:MMP1 ratio and MF in a clinically relevant disease context.

## AUTHOR CONTRIBUTIONS

S.G. conceived the study, performed data acquisition, performed statistical analyses, interpreted data, wrote the manuscript draft, and revised it. P.B. conceived the study, performed data acquisition, performed statistical analyses, interpreted data, wrote the manuscript draft, and revised it. N.B.P. performed statistical analyses and reviewed the manuscript draft. S.R. performed collagen metabolism serum biomarker measurements, discussed results and strategy, interpreted data, and revised the manuscript. S.v.H. interpreted data, discussed results and strategy, and revised the manuscript. A.F. discussed results and strategy, interpreted data, and revised the manuscript. E.M.Z. performed histological measurements and revised the manuscript. M.P. performed echocardiography and TAVR, organized the clinical trial, and revised the manuscript. G.H. is the trial's principal investigator, provided serum samples for biomarker measurements, discussed results and strategy, interpreted data, and revised the manuscript. M.S. conceived, designed, and directed the study (lead), was involved in all analyses, and wrote the manuscript. All authors have read and approved the final manuscript.

## CONFLICT OF INTEREST STATEMENT

The authors declare no conflicts of interest.

## ETHICS STATEMENT

The study was approved by the Ethics Committee of the University Medical Center Göttingen (number: 10/5/16) and complied with the Declaration of Helsinki. Written informed consent for study participation including collection of myocardial biopsies was obtained from all study participants.

## Supporting information



Supporting Information

## Data Availability

Data are available from the corresponding author upon reasonable request.
