# Supplementary material for: Estimating myocardial fibrosis in aortic stenosis using the serum collagen type I C‐terminal telopeptide to matrix metalloproteinase‐1 ratio
Source: MedComm (2020). 2025 Jan 16;6(2):e70069. doi: 10.1002/mco2.70069 (PMC11739461; doi:10.1002/mco2.70069)
Supplement: Supplementary file 1 — Supporting Information [file MCO2-6-e70069-s001.docx]

**Estimating Myocardial Fibrosis in Aortic Stenosis Using the Serum Collagen Type I C-terminal Telopeptide to Matrix Metalloproteinase-1 Ratio**

***-Supplementary Material-***

Svante Gersch^1,†^, Philipp Bengel^1,†^, Niels B. Paul^1^, Susana Ravassa^2^, Stephan von Haehling^1^, Andreas Fischer^1^, Elisabeth M. Zeisberg^1^, Miriam Puls^1^, Gerd Hasenfuß^1^, Moritz Schnelle^1,*^

^1^ Department of Clinical Chemistry, Department of Cardiology and Pneumology; University Medical Center Göttingen, Göttingen, Germany

^2^ Program of Cardiovascular Disease, Centro de Investigacion Medica Aplicada Universidad de Navarra (CIMA), Pamplona, Spain

^†^ these authors contributed equally

* corresponding author

**Correspondence to:**

Moritz Schnelle, MD, Ph.D.

Department of Clinical Chemistry, University Medical Center Göttingen

Robert-Koch-Str. 40, 37075 Göttingen, Germany

Phone: +49 (0)551 39-65510, Fax: +49 (0)551 39-63742

Email: [moritz.schnelle@med.uni-goettingen.de](mailto:moritz.schnelle@med.uni-goettingen.de)

**Supplemental Table 1:** Baseline characteristics of the study cohort. Continuous variables are expressed as mean ± standard deviation, categorical data are represented as frequency and percentage.

| **parameters** | **Total Cohort**  **(n=95)** | **NEF-HG**  **AS**  **(n=39)** | **LEF-HG**  **AS**  **(n=14)** | **Classical LF-LG**  **AS**  **(n=26)** | **PLF-LG**  **AS**  **(n=16)** |
| --- | --- | --- | --- | --- | --- |
| Age (y) | 78.4 ± 6.9 | 77.3 ± 7.2 | 77.4 ± 8.2 | 78.8 ± 6.3 | 81.0 ± 4.8 |
| Male, n (%) | 64 (67) | 25 (64) | 9 (64) | 21 (80) | 9 (56) |
| BMI (kg/m^2^) | 28.2 ± 5.7 | 28.3 ± 5.7 | 28.6 ± 7 | 27.7 ± 4.6 | 29.5 ± 5.7 |
| Diabetes mellitus, n (%) | 46 (48) | 18 (46) | 5 (35) | 15 (58) | 8 (50) |
| arterial hypertension, n (%) | 87 (91) | 38 (97) | 10 (71) | 23 (88) | 16 (100) |
| chronic coronary syndrome, n (%) | 65 (68) | 25 (64) | 9 (64) | 20 (77) | 11 (68) |
| prior CABG, n (%) | 10 (10) | 4 (10) | 1 (7) | 4 (15) | 1 (6) |
| atrial fibrillation / flutter, n (%) | 39 (41) | 13 (33) | 6 (43) | 8 (31) | 12 (75) |
| COPD, n (%) | 18 (19) | 6 (15) | 2 (14) | 7 (37) | 3 (19) |
| **Medication** | | | | | |
| Vitamin K Antagonist, n (%) | 16 (17) | 5 (13) | 4 (28) | 3 (12) | 4 (25) |
| NOAC, n (%) | 17 (18) | 5 (13) | 2 (14) | 3 (12) | 7 (43) |
| angiotensin converting enzyme inhibitor, n (%) | 50 (51) | 22 (56) | 6 (43) | 12 (46) | 10 (62) |
| Angiotensin II receptor antagonist, n (%) | 23 (24) | 10 (26) | 2 (14) | 7 (27) | 4 (25) |
| Sacubitril/Valsartan, n (%) | 3 (3) | 1 (3) | 1 (7) | 1 (4) | 0 (0) |
| betablocker, n (%) | 61 (64) | 25 (64) | 8 (57) | 17 (65) | 11 (69) |
| aldosterone antagonist, n (%) | 19 (20) | 7 (17) | 5 (35) | 6 (23) | 1 (6) |
| statine, n (%) | 58 (61) | 26 (67) | 8 (57) | 15 (58) | 9 (56) |
| oral antidiabetics, n (%) | 28 (29) | 13 (33) | 2 (14) | 9 (35) | 4 (25) |
| **Laboratory** | | | | | |
| NT-proBNP (ng/L) | 5008 ± 10025 | 1947 ± 2674 | 10786 ± 11468 | 8291 ± 15270 | 2419 ± 1210 |
| LDL (mg/dl) | 102 ± 43 | 112 ± 46 | 93 ± 33 | 96 ± 41 | 90 ± 40 |
| Cholesterol (mg/dl) | 171 ± 48 | 186 ± 51 | 156 ± 37 | 165 ± 47 | 150 ± 32 |
| Creatinine (mg/dl) | 1.34 ± 1.2 | 1.25 ± 0.9 | 1.39 ± 1.1 | 1.61 ± 1.8 | 1.17 ± 0.4 |
| eGFR (ml/min/1.73 m^2^) | 59 ± 23 | 62 ± 24 | 58 ± 20 | 54 ± 22 | 55 ± 19 |
| **Echocardiography** | | | | | |
| EF (%) | 52 ± 14 | 61 ± 5 | 34 ± 10 | 34 ± 9 | 59 ± 7 |
| LVEDD (mm) | 48 ± 8 | 44 ± 7 | 56 ± 8 | 52 ± 6 | 43 ± 5 |
| LVMI (g/m² BSA) | 172 ± 47 | 146 ± 38 | 180 ± 39 | 165 ± 41 | 127 ± 33 |
| Vmax (m/s) | 3.9 ± 0.7 | 4.4 ± 0.4 | 4.3 ± 0.5 | 3.2 ± 0.4 | 3.2 ± 0.3 |
| Pmean (mmHg) | 37 ± 15 | 48 ± 13 | 45 ± 10 | 24 ± 6 | 24 ± 5 |
| aortic valve area (cm^2^) | 0.7 ± 0.1 | 0.7 ± 0.1 | 0.6 ± 0.1 | 0.7 ± 0.2 | 0.7 ± 0.1 |
| LAVI (ml/m^2^ BSA) | 53 ± 17 | 47.9 ± 14 | 61.5 ± 25 | 50.0 ± 13 | 47.0 ± 8 |

BMI: body mass index; CABG: coronary arter bypass graft; COPD: chronic obstructive pulmonary disease; EF: ejection fraction; eGFR: estimated glomerular filtration rate; LAVI left atrial volume index; LVEDD: left ventricular end-diastolic diameter; LDL: Low Density Lipoprotein; LVMI: left ventricular mass index; NOAC: novel oral anticoagulant; NT-proBNP: B-type natriuretic peptide; Pmean: Mean transaortic gradient; Vmax: aortic valve maximal velocity; NEF-HG AS: normal ejection fraction and high transvalvular pressure gradient AS; LEF-HG AS: low/reduced ejection fraction and high transvalvular pressure gradient AS; classical LF-LG AS: classical low-flow low-gradient AS; PLF-LG AS: paradoxical low-flow low-gradient despite normal ejection fraction AS

**MATERIAL AND METHODS APPENDIX**

**Study population and specimen handling**

Our analyses were carried out in myocardial tissue and according serum samples (n=95) from a recently described, well-characterized cohort of patients with severe aortic stenosis (AS) at the University Medical Center Göttingen (UMG).^1^ Endomyocardial, ventricular biopsies were obtained during aortic valve replacement procedures, whereas serum was collected beforehand. Tissue biopsies were either snap-frozen in liquid nitrogen and stored at -80°C until required, or fixed in 10% paraformaldehyde (PFA) and paraffin-embedded for histological assessment. Serum from AS patients was collected in vacutainer tubes (Sarstedt, Germany), left in a vertical position for 30 minutes, followed by centrifugation for 10 minutes at 2.000 x g. The supernatant was then snap-frozen and stored at -80°C.

The investigation conforms with the principles outlined in the Declaration of Helsinki and was approved by the institutional ethics committee. All patients provided written informed consent regarding this study.

**Echocardiography**

Echocardiography was performed as recently described.^1^ Briefly, all echocardiograms were carried out using either a Philips ie33 or a Philips Epiq7 system, routinely recorded in a Picture Archiving and Communication System. All measurements from AS patients were retrospectively re-evaluated by a single observer using Q Station 3.8.5 (Philips healthcare). Ejection fraction (EF) was assessed by biplane method of disks, and LV mass was calculated by the ASE (American Society of Echocardiography) recommended cube formula as recommended.^2^

The following criteria were used to categorize the AS patients into the four hemodynamic subgroups:

1. Normal/preserved ejection fraction, high-gradient AS (NEF-HG AS): LVEF ≥50%, Vmax ≥4 m/s or Pmean ≥40 mmHg, AVA ≤1.0 cm²
2. Low/reduced ejection fraction, high-gradient AS (LEF-HG AS): LVEF <50%, Vmax ≥4 m/s or Pmean ≥40 mmHg, AVA ≤1.0 cm²
3. Low/reduced ejection fraction, low-gradient AS (classical low-flow low-gradient AS, classical LF-LG AS): LVEF <50%, Vmax <4 m/s, Pmean <40 mmHg, AVA ≤1.0 cm², SVI ≤35 mL/m²
4. Paradoxical low-flow low-gradient AS (PLF-LG AS): LVEF ≥50%, Vmax <4 m/s, Pmean <40 mmHg, AVA ≤1.0 cm², indexed AVA ≤0.6 cm²/m², SVI ≤35 mL/m²

**Histology**

Myocardial tissue samples were slowly brought from -80°C to -20°C to 4°C and were then PFA fixed, followed by paraffin embedding and sectioning according to standard procedures. Assessment of collagen volume fraction to assess the level of myocardial fibrosis was conducted by visualisation and quantification (using ImageJ) of collagen content detected by Masson’s Trichrome Stain (MTS) following manufacturer´s instructions (Sigma-Aldrich, USA).

**Serum biomarker assessment**

Prior to transcatheter aortic valve replacement (TAVR) procedures, serum was collected from AS patients. Serum markers of collagen metabolism, i.e. the procollagen type I C-terminal propeptid (PICP), the amino-terminal propeptide of procollagen type III (PIIINP) and the ratio of collagen type I C-terminal telopeptide to matrix metalloproteinase-1 were measured using the EIA MicroVue for PICP (Quidel Corporation, USA), a radioimmunoassay for PIIINP (OrionDiagnostica, Finland), an ELISA-based assay for CITP (Orion Diagnostica), and an alphaLISA for quantification of total serum MMP-1 levels (PerkinElmer, USA). The inter- and intra-assay coefficients of variation for PICP measurements were 11.72% and 4.54%, for PIIINP 6.86% and 6.47%, for CITP 7.40% and 7.26%, and for MMP-1 13.04% and 10.30%, respectively. Limits of detection were 0.2 ng/mL (PICP), 0.3 ng/mL (PIIINP), 0.6 ng/mL (CITP) and 0.082 ng/ml (MMP-1). CITP and MMP-1 values were expressed in molarity and the ratios were calculated. Two CITP:MMP1 and one PICP measurements had to be excluded as they were outside of the standard curve range.

**Statistics**

All analyses were performed using R version 4.3.3 in a Ubuntu 20.04.6 LTS system (section Session and Library Information). All reported p-values represent, if not explicitly mentioned, the significance levels without correction for multiple hypothesis testing, since this study represents an explorative analysis approach. Alpha-mistake probabilities below 5% were considered tolerable, so that p-values below this threshold were considered significant.

Data transformation

For the comparison of biomarker levels and fibrosis between the four hemodynamic subgroups and for the partial correlation between biomarker levels and fibrosis, the data were tested for a significant deviation from normal distribution. For this purpose the Shapiro-Wilk test *(shapiro.test() function from the R package stats)*^3^ was used. If a data set deviated significantly from a normal distribution, a transformation via base 2 logarithm was tested. If data transformation was used to diminish deviation from normal distribution, it is explicitly mentioned. in the results.

Biomarker and fibrosis differences between hemodynamic AS subgroups

In order to check for differences in biomarker levels and myocardial fibrosis between the four hemodynamic subgroups, omnibus tests were used. If the biomarker levels in the groups did not deviate significantly from a normal distribution and did not differ significantly in variance, an ANCOVA *(anova() function with the lm() function both from the R package stats){Citation}* was used to examine whether the biomarker levels differed significantly between any of the groups, controlling for age, sex and eGFR (determined using the CKD-EPI formula). If the normal distribution criteria was not met, while the variance homogeneity was not violated significantly, Kruskal-Wallis Test (kruskal.test() function from the R package stats)^3^ was utilized to examine possible differences between AS groups. In order to check for variance homogeneity, the Levene Test (leveneTest() function from the R package car)^4^ was used.

Partial correlation of biomarker levels and fibrosis

In order to examine the correlation of biomarkers with myocardial fibrosis while controlling for age, sex and the eGFR (determined using the CKD-EPI formula), the partial correlation coefficients and their significance levels were calculated. Partial correlation aims to calculate the association of a predictor variable (in this case the biomarker level) and a measured variable (in this case the myocardial fibrosis) while the removing the effect of a set of controlling variables. This allows to estimate the independent effect of the biomarker onto myocardial fibrosis. The partial correlation and its p-value were computed using the pcor() function from the R package ppcor.^5^ Whether the pearson or the spearman correlation coefficient was used for this analysis was decided by checking for significant deviations of the biomarker and fibrosis levels from the normal distribution by using the aforementioned implementation of the Shapiro-Wilk test (shapiro.test() function from the R package stats).^3^

**References**

1. Puls M, Beuthner BE, Topci R, Vogelgesang A, Bleckmann A, Sitte M, Lange T, Backhaus SJ, Schuster A, Seidler T, Kutschka I, Toischer K, Zeisberg EM, Jacobshagen C, Hasenfuß G. Impact of myocardial fibrosis on left ventricular remodelling, recovery, and outcome after transcatheter aortic valve implantation in different haemodynamic subtypes of severe aortic stenosis. European Heart Journal 2020;41:1903–1914.

2. Baumgartner H, Hung J, Bermejo J, Chambers JB, Evangelista A, Griffin BP, Iung B, Otto CM, Pellikka PA, Quiñones M. Echocardiographic Assessment of Valve Stenosis: EAE/ASE Recommendations for Clinical Practice. Journal of the American Society of Echocardiography 2009;22:1–23.

3. R Core Team, R: A Language and Environment for Statistical Computing. R Foundation for Statistical Computing, Vienna, Austria, 2024.

4. J. Fox and S. Weisberg, An R Companion to Applied Regression. Thousand Oaks CA: Sage,third ed., 2019.

5. S. Kim, ppcor: Partial and Semi-Partial (Part) Correlation, 2015. R package version 1.1.
